# Annotations

**Published:** 1890-09-06

**Authors:** 


### Annotations.

**Kleptomania.**

Probably no uncivilized man believes in kleptomania. Even among civilized peoples many persons smile incredulously when they are told of the acts of kleptomaniacs. They know better: the kleptomaniac is a thief with a fine name; and the name has been invented for the purpose of screening the higher classes who indulge in low vices. But a case has been brought to light recently which ought to convince the most sceptical. A man was arrested a short time ago in the act of stealing a pocket handkerchief from a lady in a Vienna suburb. In his sane days he had been a prosperous baker; but a mania for cambric pocket handkerchiefs seized upon him and proved his ruin. It was his habit to accost ladies in the street and offer to buy their pocket handkerchiefs. If they refused he used to get angry, and to offer higher and higher prices until a bargain was struck. Many ladies—could they have been ladies?—traded upon his madness, until at last all his money was spent, and he became a bankrupt. But bankruptcy did not cure his mania, for, no longer having money to pay for pocket handkerchiefs, he took to stealing them, and was sent to prison. For five years nothing was heard of his depredations, and it was believed that his imprisonment had cured him. But a short time ago he was discovered at his old tricks. When arrested he had fifteen cambric handkerchiefs in his possession, all of which he confessed to having stolen within an hour. In his bed-room 434 cambric pocket handkerchiefs were found, and it is believed that many more were concealed in hiding-places which he refused to reveal. He had never been known to steal anything else; nor does he seem to have made any use of the cambric handkerchiefs. The tribunal before which he appeared very properly sent him to a mad-house, and not to prison. This case is absolutely convincing; and probably no one who reads it will in future doubt the fact of kleptomania. But that it should ever have been doubted is surprising and not very creditable. The world has been familiar with the delusions of the lunatic from its earliest ages; and many of those delusions have been infinitely more curious and difficult of belief than that a man should have too keen a desire to possess himself of other people's property. It is probable, in fact, that the commonest of all forms of madness is that induced by excessive greed; and the only reason why so few persons are charged with theft or kleptomania is that their greed is tempered and kept in check by cunning. Whenever a case happens where the motive for theft is exceedingly small or entirely wanting the person who possesses himself of what is not his own should be suspected of kleptomania.

**The Cholera Scare.**

With reference to the case of cholera recently occurring in London, it was noted as follows by the Local Government Board:—"The circumstances as to the alleged case of Asiatic cholera in the Poplar Hospital have been investigated by Mr. W. H. Power of the Medical Department of the Local Government Board. The patient, who had served as a coal-trimmer on board the steamship 'Duke of Argyll,' which reached the Thames on Sunday last, the 17th inst., has been suffering from symptoms which, although clinically undistinguishable from true cholera, are from time to time observed in cases of *cholera nostras*, such as occur annually at this season of the year in London and other large cities. The 'Duke of Argyll' left Calcutta on June 21st, and did not touch at any port on her way home after she left Port Said on July 30th. Throughout the entire voyage from Calcutta up to the time when her crew left her on Sunday last no illness in any way resembling cholera had occurred on board. The patient, who was not attacked until Monday night, upwards of twenty-four hours after leaving the vessel, is now on the mend, and gives promise of recovery." Now when an ailment is "clinically undistinguishable" from true cholera, we think it would be advisable to call it cholera, on the principle of terming a spade a spade. Every authority on so-called Asiatic cholera informs us that when cholera prevails, there are numerous cases of diarrhœa, many of which develope into cholera. And of such cases no one can assert whether or not they will develop into cholera. To call a malady cholera only, when certain symptoms are present, is not in accordance with the breadth of view which prevails with regard to other maladies. No one would assert that when typhoid fever, or scarlet fever, or diphtheria, or small-pox occurs in a mild form the malady presenting should not be called by the name of the severe type. It is not sufficiently realised that there are mild and severe cases of cholera. In fact, there is scarcely any disease presenting more diverse symptoms. Every epidemic almost shows different appearances. The late Dr. Norman Chevers, of Calcutta, after seeing numerous cholera epidemics in India, stated that the last he saw presented symptoms entirely new to him. Yet our text-book writers lay down that a malady is not cholera unless there are rice-water purgings and vomitings, cramp in the legs, and suppression of urine. The question has been asked at what stage does the sufferer from diarrhœa become the victim of cholera. And this question has never been answered! As a matter of fact it cannot be answered. Cholera is supposed to be conveyed from its so-called "home" in the East. But there is every reason to believe that the disease occurs in all countries, although more severely in the Eastern tropics. Summer diarrhœa and so-called *cholera infantum* is often "clinically undistinguishable" from cholera. Summer diarrhœa has been attributed to a microbe, and so has cholera. Summer diarrhœa occurs at the period of the greatest heat, and cholera occurs in the maximum intensity in hot climates. The pathological conditions also present similarity. With regard to the recent case, it may be doubted if it were imported from the East. The incubation period rarely exceeds twelve days. It was doubtless one of those cases of the cholera of temperate climates, with which the accidental circumstance of arrival from Calcutta had nothing to do. The recognition that summer diarrhœa is really a mild phase of cholera would be beneficial as leading to increased sanitary precautions, which are the only preventive means against both complaints.

**A New Tea.**

Some of the Kent people have made up their minds that hops which make such capital beer will make equally excellent tea. They are hard at work now elaborating a process for converting hops into fresh tea leaves. The object of this is not merely to give us an entirely new tea, but to furnish us with an article which will alter both the flavour and the quality of the teas we have now in use. The hop is an admirable plant. That the most resolute total abstainer cannot deny. When its dried flower is used for the making of a fresh infusion, in which fermentation plays no part, a drink is produced which is quite free from alcohol. Such a drink, when properly made, contains the tonic, soothing, and nutritive properties of the hop without any objectionable admixture. The question of flavour remains. Hop tea alone is not particularly agreeable to the unsophisticated palate; but neither is beer or stout. And yet the majority of English people gradually acquire a very keen relish for well-brewed beer. Why should they not also learn to appreciate the flavour of hop tea? If used alone the taste of the infusion could easily be modified by the addition of cream, or sugar, or other substance. But the idea of the Kent hop growers seems to be to use the hop as a qualifier and improver of the common India and China teas. We are glad to find that the subject has been taken up in a thoroughly business-like way, and that works have been started at Maidstone whereby the hops will be submitted to the drying and rolling processes which will reduce them to the appearance of the teas so familiar to the English purchaser. In this finished state they may be used for admixture with other teas; or they may be ground to powder and mixed with coffee or cocoa. During the last week or two a great many people have visited the works at Maidstone, including Mr. C. Whitehead, Agricultural Adviser to the Privy Council, and Dr. M. A. Adams. It is much to be desired that every loyal tea merchant will interest himself in the new manufacture, and that British householders who value the prosperity of the country and their own health will give the finished product a trial. From a health point of view there is no doubt whatever of the value of hop infusion. It will be satisfactory if taste can be made to approve, as well as digestion and health.

